# The Impact of Pediatric Epilepsy on Children and Families: A Multicenter Cross-Sectional Study

**DOI:** 10.2174/1745017901814010323

**Published:** 2018-12-31

**Authors:** Ahmed Hussein Subki, Abdel Moniem Mukhtar, Rakan Salah Al-Harbi, Abdulaziz Khaled Alotaibi, Faisal Ghazi Mosaad, Mohammed Saad Alsallum, Mohammed M.S. Jan

**Affiliations:** 1 Department of Pediatrics, Faculty of Medicine, King Abdulaziz University, Jeddah, Saudi Arabia; 2 Department of Family and Community Medicine, Faculty of Medicine, King Abdulaziz University, Jeddah, Saudi Arabia

**Keywords:** Epilepsy, Child, Family, Cerebral palsy, Quality of life, Pediatric epilepsy

## Abstract

**Background and Objectives:**

Epilepsy is considered one of the most prevalent causes of morbidity in children. The aim of this study is to determine how epilepsy impacts the lives of children with epilepsy and their families.

**Methods:**

A translated version of the “Impact of Pediatric Epilepsy Scale” (IPES) questionnaire was completed by the 80 mothers of children with epilepsy, recruited at three hospitals in Jeddah, Saudi Arabia This is a validated self-administered questionnaire used to assess the impact of epilepsy on the lives of the child and family, as well as the quality of life (QoL) of the child.

**Results:**

The mean age of children epilepsy was 6.32 years (SD = 3.22). The mean IPES score was 6.28 (SD = 8.42) and the mean child’s QoL was 2.85 (SD= 0.83). 87.5% of the mothers rated their child’s QoL as low. IPES score was significantly associated with cause of seizure (β=0.259; 95%-CI= 0.263 - 10.334; p = 0.039). Child’s QoL was significantly associated with frequency of seizure (β=0.251; 95%-CI= 0.016 - 0.568; p= 0.039) and child’s nationality (β=-0.270; 95%-CI -0.252, -0.013; p= 0.031).

**Conclusions:**

Pediatric epilepsy may have a greater impact on the lives of the child and the family when it is not comorbid with cerebral palsy. Quality of life tends to be lower for non-Saudi children, and children with more frequent seizures. Therefore, these groups may need more support in managing the impact that epilepsy has on their daily functioning and quality of life.

## 1 INTRODUCTION

Epilepsy, defined as recurrent unprovoked seizures originating from abnormal electrical signals in the brain, is a common chronic neurological illness in childhood [1, 2]. Epilepsy is a highly prevalent disorder around the world with a reported frequency of four to eight cases per 1000 children [3, 4]. The prevalence of epilepsy varies by country [3], with a range extending from 0.9 to 58 per 1000 people [4-18]. Estimates for children range from 5.1 to 7 cases per 1000 [19, 20] Prevalence tends to be higher in low to middle income countries when compared to high income countries [21]. There has been limited investigation of its prevalence in Middle Eastern countries. However, one study identified a prevalence of 6.5/1000 for Saudi Arabia [18]. The data on pediatric epilepsy are even more limited, with one study from 1998 estimating prevalence at 2.5/1000 [22]. However, more recent investigation is warranted.

Epilepsy represents a significant cause of morbidity as it is considered to be the most common cause of referral to outpatient pediatric neurology clinics [1]. It is a progressive and complex disease with an unpredictable and debilitating nature. Coupled with the social stigma surrounding it and the economic burden associated with it, its effects extend not only to the individual suffering from it, but also to the family unit as a whole [23-27]. This is often characterized by stigmatization, dependence, low self-esteem, depression and emotional instability alongside social, occupational and financial restrictions [27]. In Arab populations, patients with epilepsy experience elevated levels of depression and anxiety, cognitive impairment, behavioral problems, sexual dysfunction, and underemployment [28]. Quality of life appears to be similarly impaired in both European and Middle Eastern samples [29]. Stress levels are often high in caregivers of patients with chronic diseases such as epilepsy, which can lead to lower parent–child relationship quality, a higher risk of depression in mothers, and problems with family functioning [30]. Families of children with epilepsy are more likely than families of healthy children to experience social and marital issues, impaired parent-child relationships, and higher levels of stress, depression and anxiety [30-33]. In addition, disease burden may worsen associated depression [33, 34], Mothers in particular often assume the role of primary caregivers to their children [35], and are therefore at a higher risk of developing psychological issues, *e.g.*, depression [36, 37]. It is therefore important not only to consider the effect that pediatric epilepsy has on the child, but also on other family members including caregivers and siblings.

Although both mothers and fathers of children with epilepsy experience diminished QoL when compared to parents of healthy children, mothers have been shown to score lower than fathers [38]. A prospective study on maternal depression found that up to half of mothers of children with epilepsy are at increased risk for having clinical depression [39]. In fact, even mothers of children with newly-onset epilepsy are at higher risk of clinical depression, with the child’s cognitive impairments being the strongest predictor [40]. In addition to depression, anxiety levels may also be higher in mothers of children with epilepsy [41]. Therefore, a regular assessment of maternal mental health during clinic visits for pediatric epilepsy may be warranted [40]. Maternal symptoms of depression may also have a negative impact on the child with epilepsy, as they have been shown to correlate with poorer health outcomes and psychopathology in the child [42, 43].

Despite the fact that epilepsy is a universal public health issue affecting more than 100 million individuals and families worldwide [44], contributing to 1% of the global burden of diseases and 80% in developing countries [45], there has been limited investigation as to its effects on family life, particularly in the Middle East. Given that scarcity in the literature and the importance of understanding the impact that epilepsy has not only on the patient, but also on the family as a whole, we have decided to evaluate the impact of pediatric epilepsy on children and their families, as rated by their mothers in 3 main hospitals in Jeddah, Saudi Arabia: (1) King Abdul-Aziz University Hospital, (2) Al-Aziziyah Maternity and Children's Hospital and (3) Jeddah Maternity and Children's Hospital.

## 2 METHODS

### 2.1 Design and Participants

This is a cross-sectional study. From September 2016 to March 2017 we recruited a total of 80 mothers of children with physician-diagnosed epilepsy at three hospitals in Jeddah, Saudi Arabia: King Abdul-Aziz University Hospital (KAUH), Al-Aziziyah Maternity and Children's Hospital (AMCH) and Jeddah Maternity and Children's Hospital (JMCH). To be included in the study, children had to be between 2 and 18 years old, have had a seizure in the past 6 months, diagnosed with Epilepsy by a pediatric neurologist, and the mothers must have been taking care of the child for the past 6 months at a minimum. We did not include fathers of children with epilepsy as mothers are more often the primary caregiver.

### 2.2 Measures

We used the “Impact of Pediatric Epilepsy Scale” (IPES), an epilepsy-specific self-administered questionnaire. The IPES is an 11-item scale that is made for parents to evaluate the impact of epilepsy on major aspects of family life. It assesses the impact of epilepsy on health, relationship with siblings and partners, participation in social and family activities, child’s academic achievement and self-esteem, and caregiver's hopes for their child's future. Each item is rated on severity between 0 (not at all) to 3 (a lot), with higher scores indicating a higher impact of that item. The highest possible score was 33. We categorized IPES score below the median as “low impact”; and equal or above median and “high impact”.

A visual analogue scale was also used to rate QoL between 1 (poor) and 6 (excellent). IPES has been widely used to assess the quality of life of children with epilepsy [46-50]. We conducted a forward-backward translation of the questionnaire and pre-test to develop an Arabic version of IPES. While we did not seek to validate the Arabic version of the IPES, the IPES has been validated and is used extensively to measure the impact of epilepsy on family life. Finally, we collected data on child’s age, gender, nationality and cognitive ability, mother’s education, family monthly income, the frequency of seizure and cause of the seizure.

### 2.3 Bias

In order to reduce selection bias, we recruited a consecutive sample of eligible mothers. The rate of refusal to participate was less than 5% in all study hospitals. With the purpose of reducing information bias, we measured our primary outcome, the impact of epilepsy on child’s and family life, using a validated and pretested questionnaire.

### 2.4 Ethical Considerations

This study was approved by the Institutional Review Board (IRB) of King Abdul-Aziz University Hospital and the Research Ethics Committee of King Abdulaziz University in Jeddah. Signed informed consent was obtained from all subjects who participated in this study.

### 2.5 Analysis

To describe our study population, we used frequencies and absolute numbers for categorical variables, and mean, standard deviation, median and interquartile range for continuous variables. Associations between two categorical variables were assessed using the Chi-squared test. To adjust for potential confounding variables, univariate and multivariate logistic regression models were constructed. A backward elimination procedure was used to select the variables for the regression model. Multiple linear regression was also used. For all statistical tests, a p-value of < 0.05 was defined as a level of significance, to adjust for multiple testing. We used the Statistical Package for Social Sciences (SPSS©) version 20. for data analysis.

## 3 RESULTS

Our study included 80 mothers. The mean age of children with epilepsy was 6.32 years (SD = 3.22). 61.2% (n=49) were male and 45.0% (n=36) were Saudis. 11.3% (n=9), 42.5%(n=34), and 46.2% (n=37) of the mothers were illiterate, attended school, or university, respectively. 63.8% (n= 51) of the families had a monthly income less than 5000 SAR and only 2.5% (n=2) had an income higher than 10000. 72.5% (n=58) suffered from weekly seizures and 8.8% (n=7) reported daily seizures. In 78.8% (n= 63) of the child with epilepsy, cerebral palsy was the cause of seizure, and in 16.3% (n=13) hypoxic-ischemic encephalopathy, was the cause. 86.2% (n= 69) of the children had cognitive impairment. Demographic information is summarized in Table **1**.

Most of the mothers (87.5%; n=70) rated their child’s QoL as low (less than the median of 3), with a mean rating of 2.85 (SD=0.83). 58.8% (n=47) and 25.0% (n=20) of the mothers rated their child’s quality of life as average and less than average, respectively. The mean IPES score of the participating mothers was 6.28% (SD=8.42). Most of the mothers (87.5%; n=70) had high impact IPES score 53.8% [43], defined as more than or equal to a median of 2.0. Scale responses are summarized in Table **2**.

In cross tabulation (Pearson Chi-square test for independence), we found a significant difference between mothers with high* vs.* low IPES score by cause of seizure. For example, 84.6% (n=11) of mothers whose children had Hypoxic-Ischemic Encephalopathy (HIE) showed high IPES impact score. There was no significant difference between mothers with high compared to low IPES score by child’s age, child’s gender, child’s nationality, mother’s education, family’s monthly income, frequency of seizure, or cognitive disability.

In cross tabulation (Pearson Chi-square test for independence), we found a significant difference between mothers who reported a high* vs.* low overall quality of life for their child by family income and cause of seizure. By family income, 94.1% (n = 48) of mothers in families with income < 5000 SAR reported that their children had a low quality of life, compared to 81.5% [22] mothers with income > 5000 to 10000 SAR (p < 0.001).

By cause of seizure, 92.1% (n = 58) and 92.3% (n = 12) of mothers whose children had cerebral palsy and HIE, respectively, reported their children to have lower quality of life (p < 0.001).

There was no significant difference between mothers with reported high* vs.* low overall quality of life by child’s age, child’s gender, child’s nationality, mother’s education, frequency of seizure, cause of seizure, or Cognitive disability.

Using univariate analysis, mothers of children who had HIE had almost 6 times higher IPES score than mothers whose children did not have HIE (OR= 5.677, 95% CI: 1.163 - 27.717). No significant association was found between IPES score (high* vs.* low) on one side, and child’s age, child’s gender, child’s nationality, mother’s education, family’s monthly income, frequency of seizure, or cognitive disability, on the other side.

Mothers of children who did not have cerebral palsy perceived their children to have an almost 5 times higher QoL than children with cerebral palsy (OR= 4.833, 95% CI: 1.208 - 19.341). No significant difference was found between mother’s perception of their child’s quality of life (high* vs.* low) on one hand, and child’s age, child’s gender, child’s nationality, mother’s education, family’s monthly income, frequency of seizure, or cognitive disability, on the other.

Using multivariate linear regression, mothers of non-cerebral palsy children reported a higher IPES score (higher impact) than mothers whose children had cerebral palsy (β=0.259, 95.0% CI: 0.263 - 10.334). No significant association was found between IPES score (high* vs.* low) on one side, and child’s age, child’s gender, child’s nationality, mother’s education, family’s monthly income, frequency of seizure, or cognitive disability, on the other.

Using multivariate linear regression analysis, mothers of non-Saudi children perceived their children to have lower quality of life than mothers of Saudi children (β=-0.270; 95.0%; CI: -0.252 - -0.013). Mothers whose children had less frequent seizures (monthly or more) perceived their children to have higher quality of life than mothers whose children had more frequent seizure (daily or weekly; β=0.251, 95.0% CI: 0.016 - 0.568). No significant association was found between IPES score (high* vs.* low) on one side, and child’s age, child’s gender, mother’s education, family’s monthly income, cognitive disability, or cause of seizure on the other side.

## 4 DISCUSSION

The present study examined the QoL of children with epilepsy as evaluated by their mothers, and the impact of epilepsy on family life in Saudi Arabia. In terms of impact on life, the most significant factor was the cause of the seizures, with HIE being the most impactful cause in the everyday life of the child and the mother. This can be attributed to the nature of moderate to severe HIE, which can affect several aspects of daily life, including cognitive, motor and sensory functioning. The lowest impact was observed in children with idiopathic causes. This can be partially explained by the nature of some epilepsy syndromes such Benign Rolandic Seizures, Idiopathic Generalized Seizures, and other idiopathic localization epilepsies, which tend to have a better prognosis and a relatively benign course, which would lead to a reduced impact [51]. It is important to note that epilepsy syndromes may be confused with motor stereotypies, or vice versa, and they may also coexist which may create a more complex picture of the epilepsy [52].

Mean IPES score in our study was 6.28, which is similar to other studies [46, 53], but lower than IPES scores in a UK study, which had a mean of 12.84 [54]. This indicates higher impact of epilepsy on the child and family among the UK sample than in our sample. This difference may be attributed to better home nursing services, or how family is structured in each country in terms of number of children and the support provided by distant family members. Further cross-cultural investigation is warranted to determine the experiential differences of children with epilepsy and families in different countries.

In our study, the mean QoL of children with epilepsy was 2.85 out of 6 which is lower than the mean QoL in another study (mean = 4.5) that had a mean IPES score of 6.59 which is close to ours [46]. This may indicate a worse QoL of children in our study population but a similar impact on the families. This further highlights the need for an intercultural comparison.

Both mother’s level of education and the child's age did not significantly affect QoL. Interestingly, the child's cognitive disability was found to have a low impact, a finding which contrasts to multiple studies [24, 55] which have found that intellectual disability in children with epilepsy was significantly associated with lower QoL [24]. Patients with epilepsy who also have an intellectual disability have also been found to suffer more behavioral problems in comparison to those without an intellectual disability [55]. Still, our findings are consistent with those of another study [56] which found no difference based on intellectual abilities. This conflict in the results of multiple studies may be attributable to different sample sizes, methodologies, or populations. Another possible reason may be due to cultural differences in attitudes towards intellectual disability.

Mothers of children who had epilepsy, but not cerebral palsy perceived their children to have an almost five times higher QoL than did mothers of children with both epilepsy and cerebral palsy. This may be associated with the added physical disability associated with cerebral palsy. Other reasons for a lower QoL may be related to the time and effort it takes to care for physical disabilities in addition to seizures, which can lead to unemployment and a higher economic burden. All of these factors may lead to high parental stress and family dysfunction [1, 57-59]. These stressors may eventually lead to the development of anxiety or depression in the mothers, which may lead to cognitive impairment [57, 58, 60]. A qualitative investigation comparing the experience of mothers caring for children with epilepsy both with and without cerebral palsy would be a logical next step in determining the reasons for this difference.

Income was a significant determinant of QoL. More than 94% of families with monthly income less than 5000 SAR reported a low QoL, compared to 81.5% of families with monthly income greater between 5000 and 10000 SAR. This finding is consistent with other studies showing a correlation between QoL and family income [60, 61]. Multiple factors may play into this. Monthly income is associated with parental anxiety [61]. This can negatively influence the parent’s behavior around the child [24, 42], which may decrease the child’s QoL. If severe, this can lead to negative effects on the child personality in the future [57]. Low income can also lead to depression [60]. Both depression and anxiety can either cause or be caused by low QoL. Another factor is that families with higher income may have better access to private sector healthcare, like a daycare and rehabilitation centers. Further investigation into the mental health of mothers and of children with epilepsy is needed to determine the nature of the relationship between low income and low QoL in children with epilepsy.

Mothers of non-Saudi children perceived to their children to have a lower QoL than mothers of Saudi children, which may be attributed to the healthcare system in Saudi Arabia. Governmental hospitals provide free services to citizens, while non-citizens may not have the same number of services. A more universal problem is that illegal immigrants are at increased risk of not seeking help, due to the fear that they will get deported [62, 63]. Immigrants also face additional stressors that are not applicable to citizens, such as prejudice and difficulty integrating into society, which may reduce quality of life regardless of health status. This should be explored in future research to determine whether this effect is attributable to these social factors or to variation in healthcare services received.

Mothers whose children had less frequent seizures (monthly or more) perceived their children to have a higher QoL than mothers whose children had more frequent seizures (daily or weekly). This finding is consistent with most studies [55, 64], which have found an association between seizure frequency and lower QoL. This association may have a number of explanatory variables, one being the increased levels of anxiety in both parents and patients that occur with a higher seizure frequency [65]. Patients who experience more frequent seizures are also more likely to take anti-epileptic drugs (AEDs), which are known to be associated with reduced QoL [24, 28]. Dosage may also increase with frequency, as may the number of AEDs used and compliance with AEDs, leading to more side effects which may be distressing to patients and their families [28, 66].

One of the limitations in the present study is the convenience sampling technique used, which can lead to sampling bias. Indeed, children with cerebral palsy seem to be overrepresented in our sample. It is also important to note that all measures in the present study are subject to the bias of maternal perception. For example, mothers may want to imagine they provide a good quality of life for their children, which may skew their response to this question. Reliability and the psychiatric condition of the caregivers were not assessed, in part due to the nature of our study, as the sample was taken from pediatric department; in addition, we aimed to explore the primary caregiver's subjective outlook on their quality of life and relating it to the IPES scale. Another limitation is the single measure used to describe QoL. It may be more beneficial to use a multidimensional measure of QoL as seen in other studies of epilepsy, as this may give a more accurate account of mother’s perception of a number of different areas which encompass their child’s QoL. Lastly some patient related data may have been unavailable in the filing system of the hospitals such as, statues at time of diagnosis, and severity. Others weren't available to all the patients, such as the EEG track record. Others were not collected, due to not being one of the objectives of this paper.

A more comprehensive study that involves multiple cities in Saudi Arabia may provide further insight into the unique challenges associated with pediatric epilepsy in this country. Future research should also narrow its focus by examining the impact that pediatric epilepsy has on each member of the family, rather than examining the family unit as a whole. In particular, further investigation on the effects that pediatric epilepsy has on maternal mental health is warranted, particularly in Arab nations, as this area of investigation has received limited attention. The formulation of additional measures similar to the IPES with a narrower focus on just parents or siblings may be beneficial for this purpose.

## CONCLUSION

The present study demonstrates that income, migrant status, cause and frequency of seizures are all significant factors affecting the lives of children with epilepsy and their families as perceived by their mothers. There is a need to increase healthcare support to non-citizens and those with low income to improve their quality of life. Children with more frequent seizures may also require additional support.

## Figures and Tables

**Table 1:** Demographic information.

**Variable**	**Results % (n)**
**Child Age**	–
*Mean (SD) in Years*	6.3 (3.2)
*3 years or less*	16.3% (13)
*4 - 6 years*	40.0% (32)
*7 - 9 years*	27.5% (22)
*10 years and above*	16.3% (13)
**Child Gender**	–
*Male*	61.2% (49)
*Female*	38.8% (31)
**Child Nationality**	–
*Saudi*	45.0% (36)
*Egyptian*	12.5% (10)
*Syrian*	17.5% (14)
*Somalia*	8.8% (7)
*Yamani*	7.5% (6)
*Other*	8.8% (7)
**Mother Education**	–
*Illiterate*	11.3% (9)
*School*	46.2% (37)
*University*	42.5% (34)
**Family monthly income**	–
*< 10,000 SAR*	65.0% (52)
*>= 10000 SAR*	35.0% (28)
**Family monthly income**	–
*< 5000 SAR*	63.8% (51)
*> 5000 to 10000 SAR*	33.8% (27)
*> 10000 SAR*	2.5% (2)
**Frequency of Seizure**	–
*Daily*	8.8% (7)
*Weekly*	72.5% (58)
*Monthly*	10.0% (8)
*More than monthly*	8.8% (7)
**Frequency of Seizure**	–
*Daily or Weekly*	81.3% (65)
*Monthly and More*	18.8% (15)
**Cause of Seizure**	–
*Cerebral palsy*	78.8% (63)
*Hypoxic encephalopathy ischemic*	16.3% (13)
*Unknown*	5.0% (4)
**Cognitive disability**	–
*Yes*	86.2% (69)
*No*	13.8% (11)

**Table 2:** Scale responses.

**QOL**		–	
*Low QOL (<= median)*		87.5% (70)	
*High QOL (>median)*		12.5% (10)	
*Mean (SD)*		2.85 (0.83)	
*Max – Min*		6 – 0	
*Interquartile Range (3^rd^ – 2^nd^) =*		(3 – 3) = 0	
*1*		3.8% (3)	
*2*		25.0% (20)	
*3*		58.8% (47)	
*4*		8.8% (7)	
*5*		2.5% (2)	
*6*		1.3% (1)	
**IPES**		–	
*Low impact (<median)*		46.3% (37)	
*High impact (>=median)*		53.8% (43)	
*Mean (SD)*		6.28 (8.42)	
*Max – Min*		33 – 0	
*Interquartile Range (3^rd^ – 2^nd^) =*		(11 – 2) = 9	
